# ﻿Taxonomic notes on the genus *Chlorophorus* Chevrolat, 1863 (Coleoptera, Cerambycidae), with one new synonym and four newly recorded species from China

**DOI:** 10.3897/zookeys.1214.131143

**Published:** 2024-10-01

**Authors:** Zheng-Ju Fu, Lu Chen, Zhu Li

**Affiliations:** 1 College of Plant Protection, Southwest University, Chongqing, China Southwest University Chongqing China

**Keywords:** Cerambycinae, Clytini, longhorn beetle, new records, new synonym, taxonomy

## Abstract

*Chlorophorusfainanensis* Pic, 1918 is redescribed. Four species, *C.coniperda* Holzschuh, 1992, *C.diversicolor* Holzschuh, 2016, *C.orbatus* Holzschuh, 1991 and *C.pinguis* Holzschuh, 1992 are newly reported from China. A new synonymy, *Chlorophorusarciferus* (Chevrolat, 1863) = *Chlorophorussemisinuatus* Pic, 1949, **syn. nov**. is proposed.

## ﻿Introduction

The genus *Chlorophorus* was established by Chevrolat in 1863 with the type species *Callidiumannulare* Fabricius, 1787. It is distributed in the Old World, mainly in the Oriental and Palearctic regions. It is the largest genus in the tribe Clytini, consisting of 299 species/subspecies worldwide (Tavakilian and Chevillotte 2024). Numerous taxonomists have significantly contributed to the diversity of the genus. Pic (1908, 1916, 1920, 1924, 1931, 1943, 1949, 1950) described 34 species and Gressitt and Rondon (1970) described 11 species/subspecies from Laos. Holzschuh (1984, 1989, 1991a, 1991b, 1992, 1993, 1998, 2003, 2006, 2009, 2010, 2016, 2019) described 47 taxa and Viktora (2019, 2020, 2021, 2022, 2023) described 29 taxa since the 1980s, primarily originating from Laos, Thailand, Vietnam, Nepal, India and China.

Özdikmen (2011) conducted a study using especially Turkish species to propose a subgeneric arrangement with five subgenera. Current research indicates that the genus is polyphyletic (Lee and Lee 2020; Zamoroka 2021). Zamoroka (2021) proposed five new genera based on the three mitochondrial genes *12S rRNA*, *16S rRNA* and *COI* and two nuclear genes *18S rRNA* and *28S rRNA* of 15 species, leading to the genus Sparganophorus Zamoroka, 2021, to the subgenus Viridiphorus Zamoroka, 2021 and to the subgeneric status of *Humeromaculatus* Özdikmen, 2011 and *Perderomaculatus* Özdikmen, 2011. Based on the elytral pattern of all the taxa worldwide, Özdikmen (2022) proposed 36 subgenera for the world fauna. However, Lazarev (2024) synonymized *Sparganophorus* with *Humeromaculatus* and *Viridiphorus* with *Brevenotatus*, and he treated *Perderomaculatus* as a subgenus. As Özdikmen (2022) indicated, it should be noted that the subgeneric arrangement of *Chlorophorus* remains far from its final solution. Waiting for a general agreement, the subgenera are not considered in this article.

Prior to our study, 83 species/subspecies had been recorded in China (Chen et al. 2019; Danilevsky, 2020; Tavakilian and Chevillotte 2024). In the present study, four newly recorded species are included, making a total of 87 species/subspecies recorded in China.

## ﻿Material and methods

Pictures of adult morphology are composites taken using a digital camera Canon 7D with HELICON REMOTE (HeliconSoft, Ukraine). For detailed examination, male genitalia were extracted from specimens, cleared in 10% NaOH, and stored in ethanol 75%. The male genitalia were imaged using a Leica M205A stereomicroscope.

The following collection abbreviations are used in the text.

**BMNH** The Natural History Museum, London, UK

**BPBM** Bernice Pauahi Bishop Museum, Honolulu, USA

**CCH** Collection Carolus Holzschuh, Vienna, Austria

**MNHN** Muséum National d’Histoire Naturelle, Paris, France

**OUMNH** Oxford University Museum of Natural History, Oxford, UK

**SWU** Insect Collection of Southwest University, Chongqing, China

**SYSU** Sun Yat-sen University, Guangzhou, China

## ﻿Taxonomy

### ﻿ Chlorophorus fainanensis

Taxon classificationAnimaliaColeopteraCerambycidae

Pic, 1918

BC3893B6-6BA4-56AD-94D9-EA4380C071C8

Figs 1–6 , 7

Chlorophorus fainanensis Pic, 1918: 4. TL: China, Taiwan. TD: MNHN.
Chlorophorus (Humeromaculatus) ? fainanensis Özdikmen 2022: 654, 690.

#### Specimens examined.

China • 12♂♂ 5♀♀; Anhui Province, Huangshan City, Tangkou Town, Zhaixi Village, Huangshan Wild Monkey Valley; 10–14 VII. 2014; Qiu Jianyue and Xu Hao leg. (SWU).

#### Distribution.

China (Anhui, Zhejiang, Taiwan); Japan.

#### Redescription.

**Male**, body length: 11.6–15.4 mm; humeral width: 2.6–3.3 mm.

**Female**, body length: 11.1–14.0 mm; humeral width: 2.0–3.0 mm.

Body moderately slender, female slightly stouter than male. Body black, covered with sulphur-yellowish or olive-green pubescence; antennae black with pale grayish pubescence. Pronotum with three or four markings, vague and small markings or a pair of dots on the center of disc, and a spot before middle of each side. Scutellum covered by yellowish pubescence; each elytron marked with three black markings: 1) an externally open arc commencing on humerus and extending around to outer portion of disc just before end of basal fourth; 2) a wide transverse band in the middle; and 3) a narrower band at apical fourth. Legs dark reddish-brown covered with grayish pubescence.

Head narrow, irregularly punctured; frons wider; antennae filiform and slender, reaching the basal fourth of elytra. Third antennomere slightly longer than scape and the fourth. Pronotum rounded at sides, widest before middle, l.2 times as long as wide at widest; apical margin distinctly narrower than base; disk slightly convex, coarsely punctate. Scutellum rounded apically, slightly longer than wide. Elytra 2.8 times as long as humeral width, parallel at side and narrowed towards apex; elytral apex truncate. Legs long and narrow; femora slightly club-shaped; mesofemora carinate internally; tibiae narrow and almost straight; metatarsomere 1 as long as remainder combined.

**Figures 1–6.**
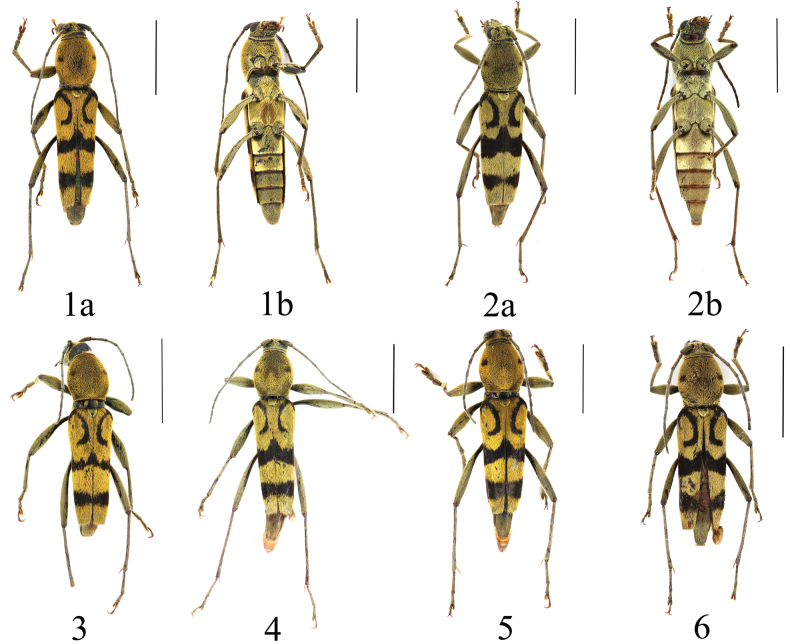
*Chlorophorusfainanensis* Pic, 1918 **1** male **a** dorsal habitus **b** ventral habitus **2** female **a** dorsal habitus **b** ventral habitus **3–5** males and **6** female from Anhui, adults, dorsal habitus. Scale bars for adult habitus: 5 mm.

**Figure 7.**
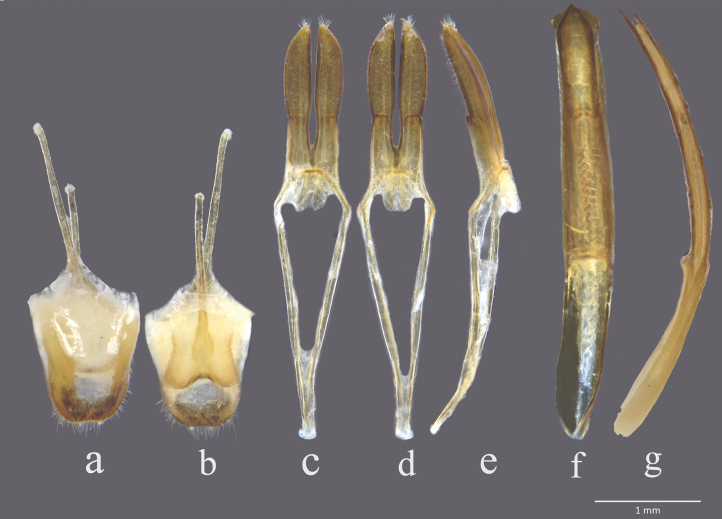
*Chlorophorusfainanensis* Pic, 1918, male genitalia **a, b** tergite VIII with sternites VIII and IX **a** dorsal view **b** ventral view **c–e** tegmen **c** dorsal view **d** ventral view **e** lateral view **f, g** median lobe **f** ventral view **g** lateral view. Scale bars for genitalia: 1 mm.

***Male genitalia*.** Tergite VIII as long as broad, apex truncate and moderately emarginate, and with long setae (Fig. 7a, b); parameres elongate, base of each paramere transversely ridged ventrally, the ridge covered with setae (Fig. 7c–e); median lobe long and slender, curved in lateral view, median struts 2/5 times as long as entire median lobe, ventral plate longer than dorsal plate, the apex of ventral plate pointed; median foramen rounded (Fig. 7f, g).

#### Remarks.

Gressitt (1951) synonymized *C.fainanensis* with *C.signaticollis* Laporte de Castelnau & Gory, 1841 (= *C.annulatus* (Hope, 1831)) and then Holzschuh (2020) resurrected it. There is a lot of variation in the pronotal and elytral markings. The species is often confused with *C.annulatus*, *C.hainanicus* Gressitt, 1940 and *C.arciferus* (Chevrolat, 1863). It can be distinguished by the preapical band on the elytra and different male genitalia: parameres 2/5 as long as the entire tegmen, neither 3/5 (*C.hainanicus* and *C.arciferus*), nor 1/4 (*C.annulatus*). It is native to Taiwan and distributed in the east of mainland China Zhejiang (Lin et al. 2023) and Anhui.

### ﻿ Chlorophorus arciferus

Taxon classificationAnimaliaColeopteraCerambycidae

(Chevrolat, 1863)

A4F8F176-8944-5AEE-A819-692EAB3711E9

Figs 8–14 , 15

Amauraesthes arciferus Chevrolat, 1863: 330. TL: India. TD: BMNH.
Caloclytus arciferus Gahan 1906: 263.
Chlorophorus arciferus Aurivillius 1912: 403.
Clytanthus varius v. *pieli* Pic, 1924:15. TL: China, Jiangsu. TD: MNHN.
Clytanthus verbasci v. *rectefasciatus* Pic, 1937: 14. TL: Vietnam. TD: MNHN.
Chlorophorus semisinuatus Pic, 1949: 54. TL: India. TD: MNHN. syn. nov.
Chlorophorus (Immaculatus) arciferus Lazarev 2019: 147.
Chlorophorus (Humeromagnomaculatus) arciferus Özdikmen 2022: 655, 687, 691.

#### Specimens examined.

China • 13♂♂ 12♀♀; Xizang, Lingzhi City, Chayu County, Shangchayu Town, Shizhong Village; 1700 m; 26 VIII. 2017; Qiu Jianyue, Peng Chenli and Xu Hao leg. (SWU) • 4♂♂ 5♀♀; Xizang, Lingzhi City, Motuo County, Beibeng Township, Yarang Village; 800 m; 21 VIII. 2017; Qiu Jianyue, Peng Chenli and Xu Hao leg. (SWU).

**Figures 8–14.**
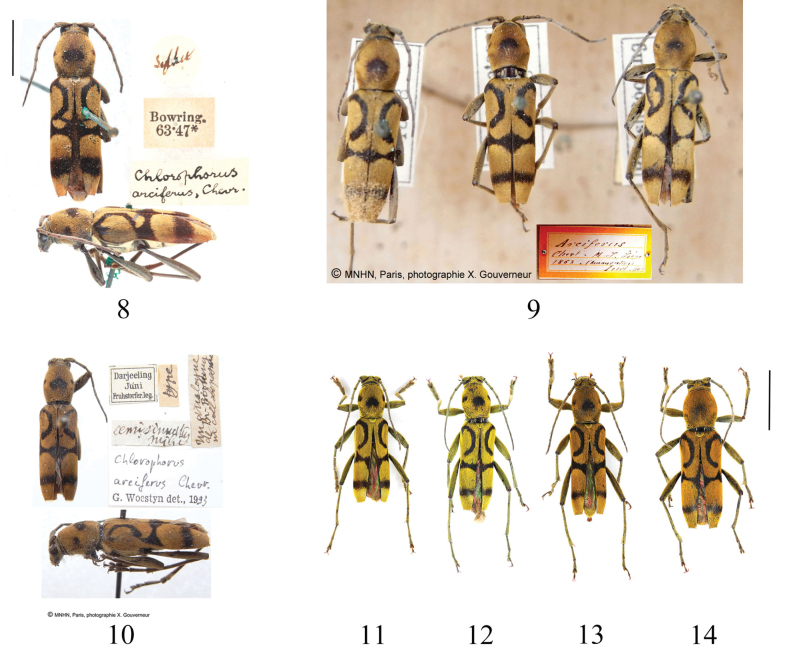
*Chlorophorusarciferus* (Chevrolat, 1863) **8** adult habitus (from BMNH photo J.-Y. Qiu and H. Xu) **9** adult habitus (from MNHN, photo X. Gouverneur) **10** holotype of *Chlorophorussemisinuatus* Pic, 1949 (from MNHN, photo X. Gouverneur) **11–13** males and **14** female from Xizang, adults, dorsal habitus. Scale bars for adult habitus: 5 mm.

#### Male genitalia.

**Figure 15.**
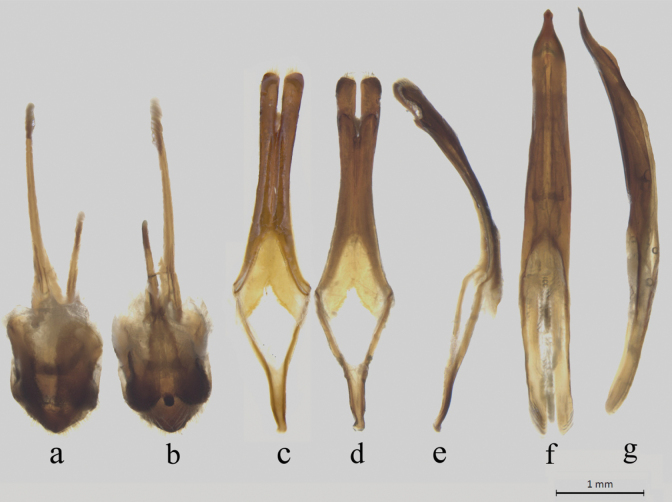
*Chlorophorusarciferus* (Chevrolat, 1863), male genitalia **a, b** tergite VIII with sternites VIII and IX **a** dorsal view **b** ventral view **c–e** tegmen **c** dorsal view **d** ventral view **e** lateral view **f, g** median lobe **f** ventral view **g** lateral view. Scale bars for genitalia: 1 mm.

Tergite VIII rounded at apical margin. Sternite VIII truncate at apical margin and with long setae (Fig. 15a, b); Tegmen weakly arcuate in lateral view, paramere 3/5 the length of tegmen, dehiscent in apical 1/4, provided with short setae near apex (Fig. 15c–e); median lobe long and slender, curved in lateral view, median struts 2/5 times as long as entire median lobe, ventral plate longer than dorsal plate, the apex of ventral plate pointed; median foramen convex (Fig. 15f, g).

#### Remarks.

Gressitt (1951) reported *C.variuspieli* from China (Shanghai, Zhejiang, Anhui, Sichuan) and treated *Clytanthusverbasci* v. *rectefasciatus* as a synonym of this taxon. Gressitt and Rondon (1970) synonymized *C.varius* v. *pieli* and *C.socius* with *C.arciferus*. However, according to Holzschuh (1991b), the figures provided in both Gressitt (1951, plate XI, fig. 5 with legend “*C.varius* v. *pieli* ?: Anhwei”) and Gressitt and Rondon (1970, fig. 36h–i; 36h marked as *C.arciferus* and 36i as “ditto subsp ?”) do not show *C.arciferus*; fig. 36h might represent *C.ictericus* Holzschuh, 1991 and fig. 36i *C.copiosus* Holzschuh, 1991. Hua (2002) reported *C.arciferus* from China (Anhui, Jiangxi, Zhejiang, Hainan, Sichuan), Laos, Bhutan, India and Nepal. But fig. 145 in Hua et al. (1993, pl. X) and fig. 334 in Hua et al. (2009, pl. XXIX) do not show *C.arciferus* either.

So far, we have examined specimens only from Xizang, China (Figs 11–14). Therefore, the distribution of this species is not wide: it might be distributed in India, Nepal, China (Xizang) and adjacent areas.

Although based on the figures of *Clytanthusvarius* v. *pieli*, Gressitt’s (1951) specimen is not *C.arciferus*. However, we did not examine the type specimen. Therefore, both are still treated as synonyms of *C.arciferus*. Furthermore, we examined the pictures of the type of *Chlorophorussemisinuatus* Pic, 1949 (Fig. 10), and found that the external morphological characteristics of this species are the same as *C.arciferus* (Figs 8–9, 11–14), so *Chlorophorusarciferus* (Chevrolat, 1863) = *Chlorophorussemisinuatus* Pic, 1949, syn. nov.

### ﻿ Chlorophorus socius

Taxon classificationAnimaliaColeopteraCerambycidae

(Gahan, 1906)

07671CB7-DD0C-5A54-B6CD-55F863A6FE36

Fig. 16

Caloclytus socius Gahan, 1906: 264. TL: India. TD: BMNH.
Chlorophorus socius Aurivillius 1912: 404.
Chlorophorus (Brevenotatus) socius Özdikmen 2022: 636, 689.

#### Specimen examined.

***Holotype***: India • 1♀; Darjeeling (BMNH).

#### Remarks.

Gressitt and Rondon (1970) synonymized *Chlorophorussocius* with *C.arciferus* and then Holzschuh (1991b) resurrected it. According to Holzschuh (1991b), *C.socius* can be separated from *C.arciferus* by having a carinate femora.

**Figure 16.**
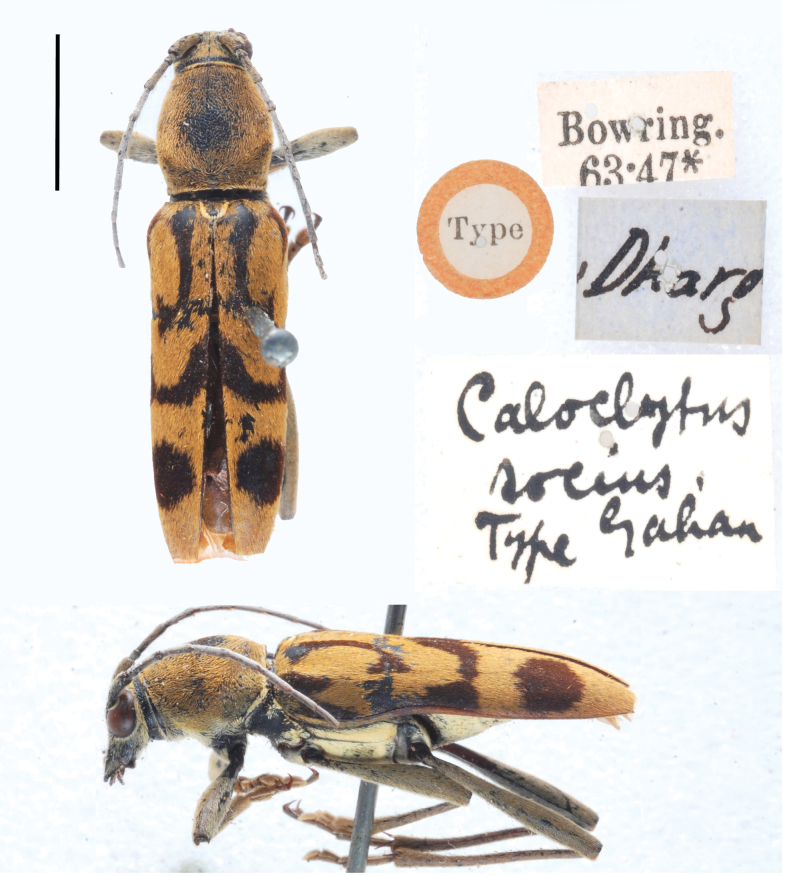
*Chlorophorussocius* (Gahan, 1906), holotype (photo J.-Y. Qiu and H. Xu). Scale bar: 5 mm.

### ﻿ Chlorophorus annulatus

Taxon classificationAnimaliaColeopteraCerambycidae

(Hope, 1831)

682536F5-A5A2-571E-9300-51D759BEFD05

Figs 17 , 18

Clytus annulatus Hope, 1831: 28. TL: Nepal. TD: OUMNH.
Clytus signaticollis Laporte de Castelnau & Gory, 1841: 103. TL: India. TD: OUMNH. Syn. by Holzschuh 2020: 49.
Anthoboscus signaticollis Chevrolat 1863: 303.
Clytanthus signaticollis Waterhouse 1874: xxviii.
Chlorophorus signaticollis Schwarzer 1925: 27.
Chlorophorus separatus Gressitt, 1940: 78. TL: China. TD: SYSU. Syn. by Holzschuh 2020: 49.
Chlorophorus nigroannulatus Pic, 1943: 1. TL: Vietnam. TD: MNHN. Syn. by Holzschuh 2020: 49.
Chlorophorus nigroannulatus v. *rufonotatus* Pic, 1943: 1. Syn. by Holzschuh, 2020: 49.
Rhaphuma signaticollis Ohbayashi 1963: 11.
Chlorophorus nigroannulatus Villiers and Chûjô 1966: 552[HN].
Chlorophorus viticis Gressitt & Rondon, 1970: 220, 225. TL: Laos. TD: BPBM. Syn. by Holzschuh 2020: 49.
Chlorophorus annulatus Holzschuh 1984: 358.
Chlorophorus (Chlorophorus) signaticollis Mitra et al. 2017: 81.
Chlorophorus (Humeromaculatus) annulatus Lazarev and Murzin 2019: 765.
Chlorophorus (Immaculatus) signaticollis Niisato in Danilevsky 2020: 231.
Chlorophorus (Chlorophorus) annulatus Özdikmen 2022: 641, 686, 690.

#### Specimens examined.

China • 16♂♂ 8♀♀; Yunnan Province, Pu’er City, Simao, Laiyang River; 11–13 V. 2018; Qiu Jianyue, Peng Chenli and Xu Hao leg. (SWU) • 25♂♂ 19♀♀; Yunnan Province, Pingbian County, Dawei Mountain; 25–27 V. 2018; Qiu Jianyue, Peng Chenli and Xu Hao leg. (SWU).

**Figures 17, 18.**
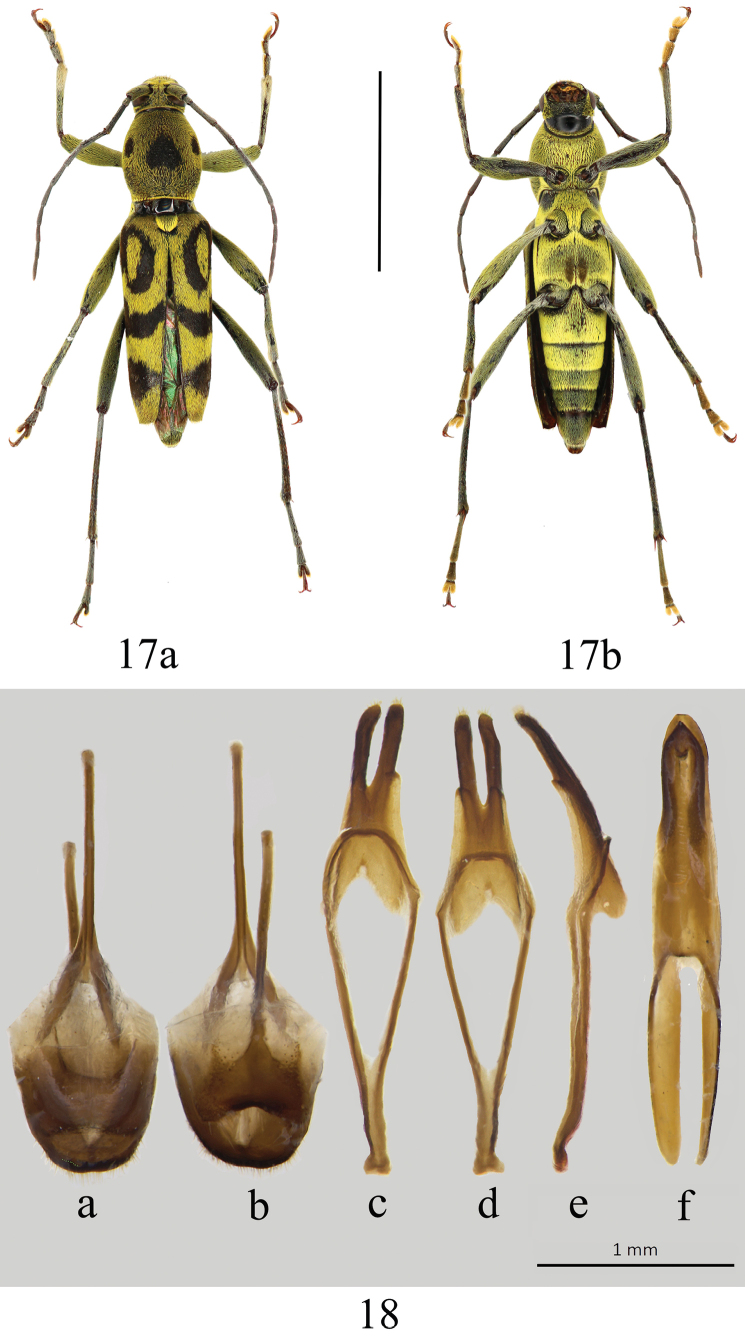
*Chlorophorusannulatus* (Hope, 1831) **17** adult habitus **a** dorsal habitus **b** ventral habitus **18** male genitalia **a, b** tergite VIII with sternites VIII and IX **a** dorsal view **b** ventral view **c–e** tegmen **c** dorsal view **d** ventral view **e** lateral view **f** median lobe, ventral view. Scale bars: 5 mm (for adult habitus); 1 mm (for genitalia).

#### Remarks.

This species is similar in elytral markings to *C.fainanensis*, but the male genitalia are distinctly different (Fig. 18).

##### ﻿New records for China

### ﻿ Chlorophorus coniperda

Taxon classificationAnimaliaColeopteraCerambycidae

Holzschuh, 1992

E5402AC8-39F9-5291-95A4-8CFA855C33B1

Fig. 19

Chlorophorus coniperda Holzschuh, 1992: 27, fig. 28. TL: Vietnam. TD: CCH.
Chlorophorus (Humeromaculatus) coniperda Özdikmen 2022: 652, 687.

#### Specimens examined.

China • 2♂♂ 1♀, Yunnan Province, Yuxi City, E’shan County; 3 V. 2021; Tian Lichao leg. (SWU) • 1♀; Yunnan Province, Xishuangbanna, Jinghong City, Dadugang; 28 IV. 2023; Tian Lichao leg. (SWU).

#### Distribution.

China (Yunnan); Vietnam.

### ﻿ Chlorophorus diversicolor

Taxon classificationAnimaliaColeopteraCerambycidae

Holzschuh, 2016

0D897B7D-B058-5AD2-AF3A-74BBCE9B693F

Fig. 20

Chlorophorus diversicolor Holzschuh, 2016: 113, figs 7, 8. TL: Laos. TD: CCH.
Chlorophorus (Humeromaculatus) diversicolor Özdikmen 2022: 652, 687.

#### Specimens examined.

China • 2♂♂ 2♀♀, Yunnan Province, Pu’er City, Ning’er, Tongxin; 4 V. 2012; Tian Lichao and Huang Guiqiang leg. (SWU).

#### Distribution.

China (Yunnan); Laos, Thailand.

#### Remarks.

There is sexual dimorphism in this species in body color. Males are light reddish-brown while females are dark reddish-brown or blackish-brown.

### ﻿ Chlorophorus orbatus

Taxon classificationAnimaliaColeopteraCerambycidae

Holzschuh 1991

F91AFA6F-03C6-5ED0-AE92-2E9C984E5A66

Fig. 21

Chlorophorus orbatus Holzschuh, 1991a: 12, fig. 11. TL: Thailand. TD: CCH.
Chlorophorus (Chlorophorus) orbatus Özdikmen 2022: 641, 686.

#### Specimens examined.

China • 2♂♂; Yunnan Province, Xishuangbanna, Jinghong City, Dadugang; 4 V. 2013; Tian Lichao leg. (SWU).

#### Distribution.

China (Yunnan); Thailand, India.

#### Remarks.

This species is similar to *C.sappho* Gressitt & Rondon, 1970. *Chlorophorussappho* can be distinguished from this species mainly by the larger body size; yellow body coloration; meso- and metafemora finely carinate externally; metatibia slightly sinuate and feebly carinate; and elytral apex toothed at the external edge.

**Figures 19–22.**
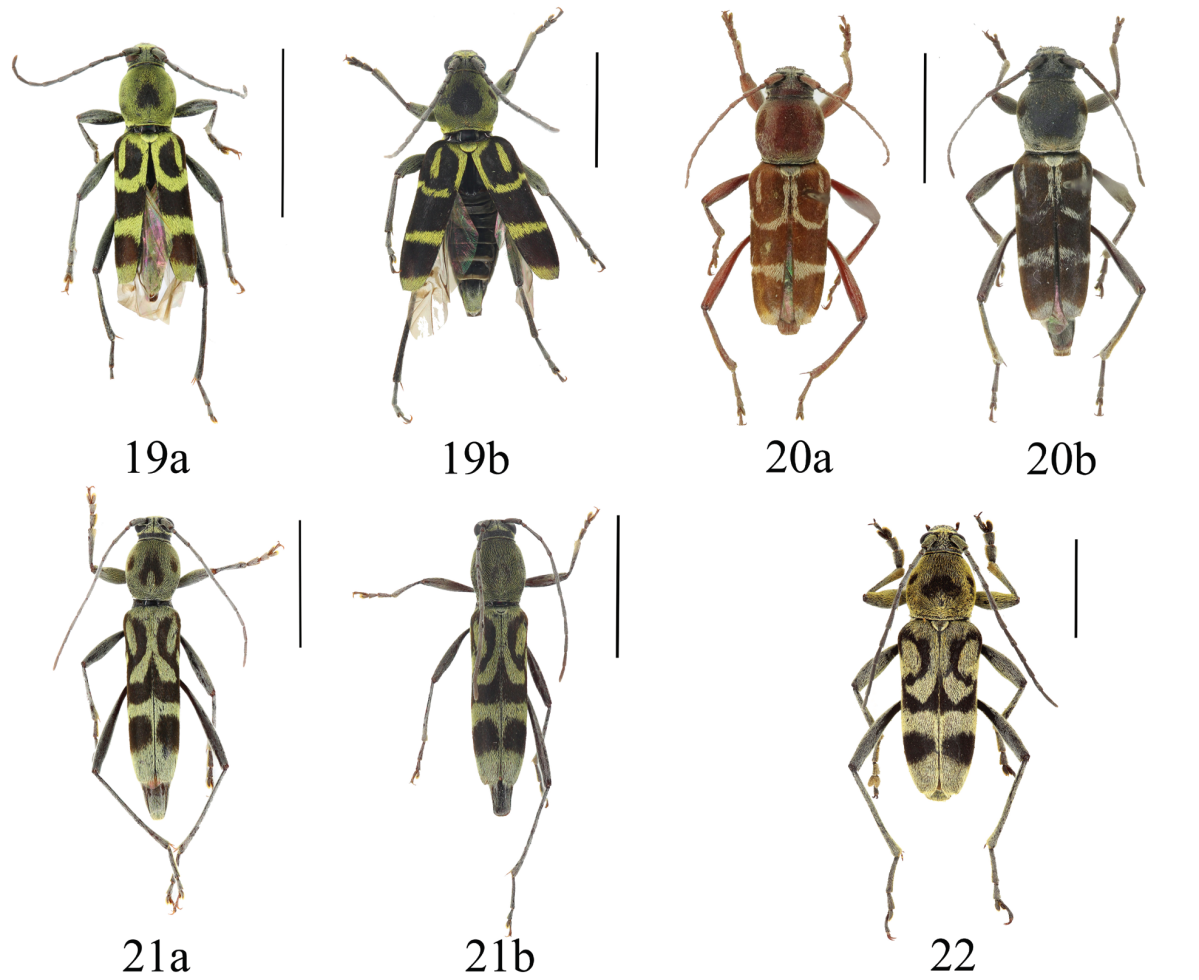
**19***Chlorophorusconiperda* Holzschuh, 1992 **a** male **b** female **20***Chlorophorusdiversicolor* Holzschuh, 2016 **a** male **b** female **21***Chlorophorusorbatus* Holzschuh, 1991 **a** male **b** female **22***Chlorophoruspinguis* Holzschuh, 1992. Scale bars for adult habitus: 5 mm.

### ﻿ Chlorophorus pinguis

Taxon classificationAnimaliaColeopteraCerambycidae

Holzschuh, 1992

8C53C55F-A933-5496-9439-32F42B16E865

Fig. 22

Chlorophorus pinguis Holzschuh, 1992: 21, figs 21, 63. TL: Vietnam. TD: CCH.
Chlorophorus (Humeromagnomaculatus) pinguis Özdikmen 2022: 655, 687.

#### Specimens examined.

China • 3♂♂ 2♀♀; Guangxi Province, Baise City, Leye County, Yachang Town; V. 2016; native collector leg. (SWU).

#### Distribution.

China (Guangxi); Vietnam.

## Supplementary Material

XML Treatment for Chlorophorus fainanensis

XML Treatment for Chlorophorus arciferus

XML Treatment for Chlorophorus socius

XML Treatment for Chlorophorus annulatus

XML Treatment for Chlorophorus coniperda

XML Treatment for Chlorophorus diversicolor

XML Treatment for Chlorophorus orbatus

XML Treatment for Chlorophorus pinguis

## ﻿Additional information

### Author ORCIDs

Zhu Li https://orcid.org/0000-0002-7322-5863

### Data availability

All of the data that support the findings of this study are available in the main text.
